# Osteoprotegerin and breast cancer risk by hormone receptor subtype: a nested case-control study in the EPIC cohort

**DOI:** 10.1186/s12916-017-0786-8

**Published:** 2017-02-08

**Authors:** Renée T. Fortner, Danja Sarink, Helena Schock, Theron Johnson, Anne Tjønneland, Anja Olsen, Kim Overvad, Aurélie Affret, Mathilde His, Marie-Christine Boutron-Ruault, Heiner Boeing, Antonia Trichopoulou, Androniki Naska, Philippos Orfanos, Domenico Palli, Sabina Sieri, Amalia Mattiello, Rosario Tumino, Fulvio Ricceri, H. Bas Bueno-de-Mesquita, Petra H. M. Peeters, Carla H. Van Gils, Elisabete Weiderpass, Eiliv Lund, J. Ramón Quirós, Antonio Agudo, Maria-José Sánchez, María-Dolores Chirlaque, Eva Ardanaz, Miren Dorronsoro, Tim Key, Kay-Tee Khaw, Sabina Rinaldi, Laure Dossus, Marc Gunter, Melissa A. Merritt, Elio Riboli, Rudolf Kaaks

**Affiliations:** 1 Division of Cancer Epidemiology, German Cancer Research Center (DKFZ), Im Neuenheimer Feld 280, D-69120 Heidelberg, Germany; 2 Diet, Genes and Environment, Danish Cancer Society Research Center, Copenhagen, Denmark; 3 Section for Epidemiology, Department of Public Health, Aarhus University, Aarhus, Denmark; 4 Université Paris-Saclay, Université Paris-Sud, UVSQ, CESP, INSERM, Villejuif, France; 5 Gustave Roussy, F-94805 Villejuif, France; 6 Department of Epidemiology, German Institute of Human Nutrition Potsdam-Rehbruecke, Nuthetal, Germany; 7 Hellenic Health Foundation, Athens, Greece; 8 WHO Collaborating Center for Nutrition and Health, Unit of Nutritional Epidemiology and Nutrition in Public Health, Department of Hygiene, Epidemiology and Medical Statistics, University of Athens Medical School, Athens, Greece; 9 Cancer Risk Factors and Life-Style Epidemiology Unit, Cancer Research and Prevention Institute – ISPO, Florence, Italy; 10 Epidemiology and Prevention Unit, Department of Preventive & Predictive Medicine Fondazione IRCCS Istituto Nazionale dei Tumori, Milan, Italy; 11 Dipartimento di Medicina Clinica e Chirurgia, Federico II University, Naples, Italy; 12 Cancer Registry and Histopathology Unit, “Civic - M.p.Arezzo” Hospital, ASP Ragusa, Italy; 13 Unit of Epidemiology, Regional Health Service ASL TO3, Grugliasco (TO), Italy; 14 Unit of Cancer Epidemiology, Department of Medical Sciences, University of Turin, Turin, Italy; 15 Department for Determinants of Chronic Diseases (DCD), National Institute for Public Health and the Environment (RIVM), Bilthoven, The Netherlands; 16 Department of Epidemiology and Biostatistics, School of Public Health, Imperial College London, London, UK; 17 Department of Social & Preventive Medicine, Faculty of Medicine, University of Malaya, Kuala Lumpur, Malaysia; 18 Department of Epidemiology, Julius Center for Health Sciences and Primary Care, University Medical Center Utrecht, Utrecht, Netherlands; 19 MRC-PHE Centre for Environment and Health, Department of Epidemiology and Biostatistics, School of Public Health, Imperial College, London, UK; 20 Department of Community Medicine, Faculty of Health Sciences, University of Tromsø, The Arctic University of Norway, Tromsø, Norway; 21 Department of Research, Cancer Registry of Norway, Institute of Population-Based Cancer Research, Oslo, Norway; 22 Department of Medical Epidemiology and Biostatistics, Karolinska Institutet, Stockholm, Sweden; 23 Genetic Epidemiology Group, Folkhälsan Research Center, Helsinki, Finland; 24 Public Health Directorate, Asturias, Spain; 25 Unit of Nutrition and Cancer. Cancer Epidemiology Research Program. Catalan Institute of Oncology-IDIBELL. L’Hospitalet de Llobregat, Barcelona, Spain; 26 Escuela Andaluza de Salud Pública. Instituto de Investigación Biosanitaria ibs. GRANADA. Hospitales Universitarios de Granada/Universidad de Granada, Granada, Spain; 27 CIBER de Epidemiología y Salud Pública (CIBERESP), Madrid, Spain; 28 Department of Epidemiology, Regional Health Council, IMIB-Arrixaca, Murcia, Spain; 29 Department of Health and Social Sciences, Universidad de Murcia, Murcia, Spain; 30 Navarra Public Health Institute, Pamplona, Spain; 31 IdiSNA, Navarra Institute for Health Research, Pamplona, Spain; 32 Public Health Direction and Biodonostia Research Institute CIBERESP, Basque Regional Health Department, San Sebastian, Spain; 33 Nuffield Department of Population Health, University of Oxford, Oxford, UK; 34 Cancer Epidemiology Unit, University of Cambridge, Cambridge, UK; 35 International Agency for Research on Cancer, Lyon, France

**Keywords:** Breast cancer, Osteoprotegerin, RANK axis, Hormone receptor, Estrogen receptor, Progesterone receptor

## Abstract

**Background:**

Circulating osteoprotegerin (OPG), a member of the receptor activator of nuclear factor kappa-B (RANK) axis, may influence breast cancer risk via its role as the decoy receptor for both the RANK ligand (RANKL) and tumor necrosis factor-related apoptosis-inducing ligand (TRAIL). Circulating OPG and breast cancer risk has been examined in only one prior study.

**Methods:**

A case-control study was nested in the European Prospective Investigation into Cancer and Nutrition (EPIC) cohort. A total of 2008 incident invasive breast cancer cases (estrogen receptor (ER)+, *n* = 1622; ER–, *n* = 386), matched 1:1 to controls, were included in the analysis. Women were predominantly postmenopausal at blood collection (77%); postmenopausal women included users and non-users of postmenopausal hormone therapy (HT). Serum OPG was quantified with an electrochemiluminescence assay. Relative risks (RRs) and 95% confidence intervals (CIs) were calculated using conditional logistic regression.

**Results:**

The associations between OPG and ER+ and ER– breast cancer differed significantly. Higher concentrations of OPG were associated with increased risk of ER– breast cancer (top vs. bottom tertile RR = 1.93 [95% CI 1.24–3.02]; *p*_trend_ = 0.03). We observed a suggestive inverse association for ER+ disease overall and among women premenopausal at blood collection. Results for ER– disease did not differ by menopausal status at blood collection (*p*_het_ = 0.97), and we observed no heterogeneity by HT use at blood collection (*p*_het_ ≥ 0.43) or age at breast cancer diagnosis (*p*_het_ ≥ 0.30).

**Conclusions:**

This study provides the first prospective data on OPG and breast cancer risk by hormone receptor subtype. High circulating OPG may represent a novel risk factor for ER– breast cancer.

**Electronic supplementary material:**

The online version of this article (doi:10.1186/s12916-017-0786-8) contains supplementary material, which is available to authorized users.

## Background

Osteoprotegerin (OPG) is a homodimeric glycoprotein and a member of the tumor necrosis factor (TNF) receptor superfamily [1]. OPG was first characterized as a negative regulator of bone turnover. Bone homeostasis is maintained by the interplay between the receptor activator of nuclear factor kappa-B (RANK), its soluble activation ligand (RANKL), and OPG. OPG binds RANKL as a decoy receptor, inhibiting the activation of RANK by RANKL and preventing the differentiation of bone marrow precursor (monocyte/macrophage) cells to osteoclasts — cells that are central in the process of bone resorption [2].

Besides bone, OPG is expressed in other tissues, including the stomach, intestines, skin, liver, heart, lung, kidney, and breast. This expression across diverse tissue types indicates that its biologic functions may extend beyond bone metabolism. In relation to breast cancer, OPG was initially investigated as a potential inhibitor of metastasis-related osteolysis [3, 4]. The RANKL inhibitor denosumab acts to reduce skeletal-related events in patients with bone metastases. More recent data show that OPG is also produced in breast tumor cells, and that it can promote tumor growth and metastasis [5, 6]. In vitro studies suggest that OPG, again acting as a decoy receptor, also binds to TNF-related apoptosis-inducing ligand (TRAIL), thereby preventing cancer cell death via apoptosis [7, 8]. Furthermore, OPG can induce cell proliferation and angiogenesis through a variety of cell surface receptors [5, 6].

Besides local expression in many tissue types, OPG is present in blood. Circulating OPG has been examined in the context of chronic disease risk, including impaired glucose tolerance and type 2 diabetes [9], vascular calcification, atherosclerosis and cardiovascular disease [10, 11], cancer [12], and overall mortality [10]. However, to date, only one prospective study has examined the relationship between circulating OPG and breast cancer risk [12]. This study observed reduced breast cancer risk among women with comparatively high OPG concentrations, but given a small number of incident cases (*n* = 76), risk by tumor subtypes (e.g., by estrogen (ER) and progesterone (PR) receptor status) was not investigated. We provide the first prospective data on OPG and breast cancer risk by hormone receptor status, in a large nested case-control study in the European Prospective Investigation into Cancer and Nutrition (EPIC) cohort.

## Methods

The EPIC cohort was designed to identify risk factors for cancer [13]. Briefly, between 1992 and 2000 about 520,000 apparently healthy men and women (*n* = 367,993 women), from 10 European countries (Denmark, France, Germany, Greece, Italy, the Netherlands, Norway, Spain, Sweden, and the United Kingdom), generally between ages 35 and 75 years, enrolled in the study. Detailed dietary, anthropometric, lifestyle, and medical history data were collected via standardized methods. Blood samples were collected according to standardized protocols (*n* = 235,607 women). Samples for this study were stored centrally under liquid nitrogen (–196 °C) at the International Agency for Research on Cancer (IARC), except those from Denmark, which were stored locally at –150 °C.

Incident cancers were identified through linkages with regional cancer registries except in France, Germany, Greece, and Naples (Italy), where follow-up is conducted by review of health insurance records, contacts with cancer and pathology registries, and/or direct contact with cohort members. Vital status is ascertained through linkages with regional and national mortality registries and active follow-up (Germany and Greece). Details on the identification and verification of incident breast cancer cases and collection of hormone receptor status data were reported previously [14].

### Nested case-control study

Case and control selection has been described previously [15]. Cases and matched controls were women either pre- or postmenopausal at the time of blood collection, with no previous diagnosis of invasive cancer (with the exception of non-melanoma skin cancer) or in situ breast cancer. Premenopausal women included in this study were not using oral contraceptives (OCs), whereas the postmenopausal study population included both hormone therapy (HT) users and non-users. Participants from Sweden were not included in this investigation, given the independent studies on breast cancer risk conducted in those centers.

The end of follow-up for this study was the last date of complete follow-up for both cancer incidence and vital status (range: 2003–2006, depending on the study center). All incident cases with ER status available were included through 2004. After 2004, all newly diagnosed ER– cases were included, and among postmenopausal women an equal number of ER+ cases were randomly selected among cases matching each ER– case for recruitment center.

One control per case was selected among cohort members who donated a blood sample and who were alive and free of cancer (except non-melanoma skin cancer) at the time of invasive breast cancer diagnosis of the index case. Controls were matched to cases on recruitment center, age (±3 months), menopausal status, HT use, fasting status (<3, 3–6, >6 hours), and time of day (±1 hour) at blood donation. Premenopausal women were also matched for phase of menstrual cycle (early follicular, late follicular, periovulatory, early luteal, mid-luteal, late luteal).

A total of 2020 case-control sets were initially selected. After excluding sets in which the case or control was missing the OPG measurement (*n* = 7 sets) or had OPG values identified as outliers (*n* = 5 sets), a total of 2008 case-control sets remained for analysis. PR status was available for a total of 74% of cases (*n* = 1480).

### Within-person reproducibility study

The EPIC-Heidelberg cohort was recruited between 1994 and 1998. The EPIC-Heidelberg substudy has been described previously [16]. Briefly, between 2010 and 2013, approximately 600 cohort participants (50% women) provided additional blood samples, with a total of 592 participants providing samples and exposure data at baseline and 14 and 15 years after recruitment. OPG was measured in repeat blood samples from 221 women randomly selected from this population to assess within-person reproducibility over 1 year (between years 14 and 15) and over 14 years (between baseline and year 14) (Additional file 1: Table S1).

### Laboratory methods

Serum OPG concentrations were measured in the Laboratory of the Division of Cancer Epidemiology at the German Cancer Research Center (DKFZ), using an electrochemiluminescence assay (MesoScale Diagnostics, Rockville, MD, USA). Samples from cases and their matched controls were analyzed in the same analytical batch, and laboratory personnel were blinded to the case-control status of the samples. The precision of the laboratory work was monitored by inclusion of blinded pooled quality control (QC) samples (two per batch). Intra-batch coefficients of variation (CVs) were 15%; inter-batch CVs were 17%.

Serum concentrations of estradiol, estrone, testosterone, dehydroepiandrosterone sulfate (DHEAS), sex hormone-binding globulin (SHBG), progesterone, prolactin, and insulin-like growth factor 1 (IGF-1) were available for subsets of the study population and were assessed as covariates and effect modifiers. Laboratory methods have been described previously [14, 15, 17, 18].

### Statistical analyses

OPG was log2-transformed to obtain an approximately normal distribution; this transformation allows an estimation of the effect of a doubling of biomarker concentration. Outliers were evaluated using the extreme Studentized deviate many-outlier procedure [19].

Spearman correlations were used to evaluate associations between OPG and continuous covariates and endogenous hormones; geometric means of OPG were compared between categories of categorical covariates. These analyses were restricted to controls and adjusted for matching factors. OPG was classified into tertiles based on the distribution in controls. As OPG levels do not vary significantly by menopausal status, tertile cut-off points were calculated based on the overall control population. Conditional logistic regression was used to estimate the relative risk (RRs) and 95% confidence intervals (CIs). Tests for trend were based on OPG modeled as a continuous variable. OPG concentrations are known to increase with age; therefore, Spearman correlations were used to examine the reproducibility of the relative ranking of study subjects over time, comparing concentrations in samples taken 1 and 14 years apart.

Ages at menarche, menopause, and first full-term pregnancy (FTP), number of FTPs, and body mass index (BMI; kg/m^2^) were included in the multivariable model. Additional adjustment for lifetime mean alcohol consumption, past oral contraceptive use, breastfeeding, and smoking status did not materially impact the results (RR_log2_ change <10%).

We evaluated heterogeneity by reproductive and lifestyle factors or endogenous hormone levels by comparing models including and excluding interaction terms using the likelihood ratio test (LRT). Tests for interaction were based on continuous, log2-transformed OPG concentrations. We assessed potential heterogeneity by tumor hormone receptor status and age at diagnosis (<50 vs. 50+ years) by comparing models assuming the same association between OPG and breast cancer in subgroups (e.g., ER+ and ER–) to one assuming different associations by subgroup using the LRT [20]. We examined the possibility of non-linearity non-parametrically with restricted cubic splines [21]. Tests for non-linearity used the LRT, comparing the model with only the linear term to the model with the linear and the cubic spline terms. There was no evidence of deviations from linearity (*p* > 0.12), with the exception of the association between OPG and ER+ breast cancer among women premenopausal at blood collection (*p* = 0.03). Finally, we performed sensitivity analyses after excluding breast cancer cases diagnosed within 2 years of blood collection (*n* = 370, 18%) to address potential reverse causation.

All statistical tests were two-tailed and significant at the *p* < 0.05 level. SAS 9.3 (SAS Institute Inc., Cary, NC, USA) was used for all statistical analyses.

## Results

A total of 462 (23%) case-control pairs were premenopausal at blood donation, whereas 1546 pairs were postmenopausal (Table 1). Among postmenopausal women, a total of 755 pairs (49%) were using exogenous hormones at blood collection. Median age at baseline was 57 (10^th^–90^th^ percentiles: 45–64) years, and among cases the median age at diagnosis was 61 (10^th^–90^th^ percentiles: 50–70) years. Median time between blood collection and diagnosis was 4.7 (10^th^–90^th^ percentiles: 1.2–8.1) years. Of the 2008 total cases, 81% were ER+ (*n* = 1622; ER–, *n* = 386).

**Table 1:** Baseline and case characteristics: EPIC cohort breast cancer nested case-control study

	Cases	Controls
*Full study population, n*	2008	2008
*Baseline characteristics, median (10* ^*th*^ *–90* ^*th*^ *percentiles) or n (%)*		
Age at blood collection, years	57 (45-64)	57 (45-64)
Age at menarche, years	13 (11-15)	13 (11-15)
Premenopausal	462 (23%)	462 (23%)
Postmenopausal	1546 (77%)	1546 (77%)
HT use at blood collection^a^	755 (49%)	755 (49%)
Age at menopause, years^a^	50 (43-55)	50 (43-55)
Completed term pregnancy	1695 (84%)	1733 (86%)
Age at first term pregnancy,^b^ years	25 (20-31)	24 (20-30)
BMI, kg/m^2^	24 (21-31)	24 (20-30)
*Case characteristics*		
ER+	1622 (81%)	
ER–	386 (19%)	
ER+/PR + ^c^	929 (63%)	
ER–/PR–	258 (17%)	
Age at diagnosis, years	61 (50-70)	
Time between blood donation and diagnosis, years	4.7 (1.2-8.1)	
*Osteoprotegerin concentrations, ng/mL, median (10* ^*th*^ *–90* ^*th*^ *percentiles)*		
	0.196 (0.138-0.282)	0.197 (0.141-0.289)

^a^Among postmenopausal women

^b^Among women with completed term pregnancy

^c^PR status available for 74% of cases (*n* = 1480); percentages represent percentage of total cases with ER and PR status available

Serum OPG concentrations were positively correlated with age (*r* = 0.29, *p* < 0.01). Among premenopausal women, OPG did not vary by menstrual cycle phase (*p* = 0.29). OPG concentrations did not vary by menopausal status at blood collection (*p* = 0.31). However, among women postmenopausal at blood collection, OPG levels differed between current users and non-users of HT (at time of blood collection; *p* = 0.01), although the absolute differences were small (adjusted geometric mean concentrations, ng/mL, HT non-users: 0.202 [95% CI: 0.197–0.207] vs. HT users: 0.210 [0.204–0.216]). Among HT users, OPG levels did not further differ by type of HT (i.e., unopposed vs. opposed estrogens; *p* = 0.64).

OPG concentrations were not correlated with age at menopause, number of FTPs, BMI (kg/m^2^), or alcohol consumption (g/day), nor did concentrations differ between women with and without completed FTP (data not shown). Higher OPG concentrations were observed in women reporting ever OC use, as compared to never users (ng/mL, OC use, never: 0.192 [0.188–0.198]; ever OC users: 0.198 [0.193–0.203]; *p* = 0.02). Among women with additional hormone measurements available (*n*: 297–2008) OPG was not correlated (*r* < |0.14|) with endogenous sex steroid hormones, IGF-I, or prolactin, among either pre- or postmenopausal women, or within strata of postmenopausal HT users and non-users (Additional file 1: Table S2).

We observed significant heterogeneity in the association between OPG and risk of ER+ vs. ER– tumors (*p*_het_ = 0.02) (Table 2). Higher concentrations of OPG were associated with increased risk of ER– breast cancer (top vs. bottom tertile RR =1.93 [95% CI 1.24–3.02]; *p*_trend_ = 0.03). In contrast, we observed a suggestive inverse association between OPG and ER+ disease (top vs. bottom tertile RR = 0.84 [95% CI 0.68–1.04]; *p*_trend_ = 0.18). The association between OPG and ER– disease did not differ by menopausal status (*p*_het_ = 0.97). However, we observed suggestive heterogeneity by menopausal status for ER+ disease (*p*_het_ = 0.05; premenopausal, RR_log2_ = 0.53 [0.31–0.88]); postmenopausal, RR_log2_ = 0.97 [0.75–1.25]; Table 3). However, there was evidence of potential non-linearity of the association between OPG and ER+ breast cancer among women premenopausal at blood collection (*p* = 0.03; Additional file 1: Figure S1), and this association should be interpreted cautiously. We observed no heterogeneity by HT use (among postmenopausal women; *p*_het_ ≥ 0.43) or by age at breast cancer diagnosis (age <50 vs. 50+; *p*_het_ ≥ 0.30; Table 4). Results were similar in analyses considering both ER and PR status.

**Table 2:** Circulating concentrations of osteoprotegerin and breast cancer risk by hormone-receptor subtype: EPIC cohort breast cancer nested case-control study

	Tertiles					
	1	2	3			
Cut points (ng/mL)	<0.18	0.18 - <0.22	≥0.22	*p*_trend_^a^	RR_log2_^b^	*p*_het_^c^
ER+/PR+						
Cases/controls	342/312	297/308	290/309			
RR (95% CI)	Ref.	0.84 (0.65-1.07)	0.81 (0.61-1.08)	0.27	0.85 (0.63-1.14)	0.02
ER+						
Cases/controls	559/510	519/544	544/568			
RR (95% CI)	Ref.	0.84 (0.70-1.02)	0.84 (0.68-1.04)	0.18	0.86 (0.68-1.08)	0.02
ER–/PR–						
Cases/controls	82/96	78/96	98/66			
RR (95% CI)	Ref.	1.12 (0.68-1.83)	2.39 (1.35-4.23)	0.04	1.89 (1.03-3.47)	
ER–						
Cases/controls	139/160	117/125	130/101			
RR (95% CI)	Ref.	1.22 (0.83-1.83)	1.93 (1.24-3.02)	0.03	1.69 (1.05-2.74)	

Conditional logistic regression models adjusted for ages at menarche (≤12, 13, 14, ≥15, missing), menopause (<44, 44-47, 48-50, 51-52, 53-54, ≥55, missing), first full-term pregnancy (no FTP, <25, 25-30, ≥30, missing), number of full-term pregnancies (0, 1, 2, ≥3, missing), and BMI (kg/m^2^, continuous)

^a^ *p*_trend_ based on log2-transformed OPG concentration

^b^RR for a one-unit change in log2-transformed OPG

^c^ *p*_heterogeneity_ comparing ER+/PR+ to ER–/PR– and ER+ to ER– subtypes, based on log2-transformed OPG concentration

**Table 3:** Circulating concentrations of osteoprotegerin and breast cancer risk by menopausal status at blood collection: EPIC cohort breast cancer nested case-control study

	Tertiles					
	1	2	3			
Cut points (ng/mL)	<0.18	0.18 - <0.22	≥0.22	*p*_trend_^a^	RR_log2_^c^	*p*_het_^d^
Hormone receptor positive cases						
*Premenopausal*						
ER+/PR+						
Cases/controls	168/152	61/77	24/24			
RR (95% CI)	Ref.	0.67 (0.43-1.04)	0.89 (0.42-1.87)	0.07	0.58 (0.32-1.05)	0.36
ER+						
Cases/controls	236/200	79/118	37/34			
RR (95% CI)	Ref.	0.55 (0.38-0.81)	0.85 (0.47-1.55)	0.01^b^	*0.53 (0.31-0.88)* ^b^	*0.05* ^b^
*Postmenopausal*						
ER+/PR+						
Cases/controls	174/160	236/231	266/285			
RR (95% CI)	Ref.	0.92 (0.68-1.24)	0.85 (0.61-1.17)	0.73	0.94 (0.66-1.33)	
ER+						
Cases/controls	323/310	440/426	507/534			
RR (95% CI)	Ref.	0.98 (0.78-1.22)	0.91 (0.72-1.15)	0.79	0.97 (0.75-1.25)	
Hormone receptor negative cases						
*Premenopausal*						
ER–/PR–						
Cases/controls	35/44	18/17	14/6			
RR (95% CI)	Ref.	1.60 (0.57-4.44)	3.77 (0.86-16.4)	0.25	2.01 (0.60-6.70)	0.87
ER–						
Cases/controls	59/70	30/30	21/10			
RR (95% CI)	Ref.	1.42 (0.67-2.99)	3.21 (1.17-8.85)	0.26	1.63 (0.70-3.84)	0.97
*Postmenopausal*						
ER–/PR–						
Cases/controls	47/52	60/79	84/60			
RR (95% CI)	Ref.	1.02 (0.55-1.89)	2.03 (1.03-3.98)	0.14	1.76 (0.82-3.77)	
ER–						
Cases/controls	80/90	87/95	109/91	0.12	1.62 (0.88-2.96)	
RR (95% CI)	Ref.	1.14 (0.68-1.90)	1.60 (0.95-2.69)			

Conditional logistic regression models adjusted for ages at menarche (≤12, 13, 14, ≥15, missing), menopause (<44, 44-47, 48-50, 51-52, 53-54, ≥55, missing), first full-term pregnancy (no FTP, <25, 25-30, ≥30, missing), number of full-term pregnancies (0, 1, 2, ≥3, missing), and BMI (kg/m^2^, continuous)

^a^ *p*_trend_ based on log2-transformed continuous variable

^b^Evidence of non-linear association, *p* = 0.03; see Additional file 1: Figure S1

^c^RR for a one-unit change in log2-transformed OPG

^d^ *p*_heterogeneity_ comparing women premenopausal to postmenopausal at blood collection based on RRlog2; among postmenopausal women, results did not differ by HT use at blood collection (*p* ≥ 0.43)

**Table 4:** Circulating concentrations of osteoprotegerin and breast cancer risk by age at diagnosis: EPIC cohort breast cancer nested case-control study

	Tertiles						
	1	2	3				
Cut points (ng/mL)	<0.18	0.18 - <0.22	≥0.22	*p*_trend_^a^	RR_log2_^b^	*p*_het_^c^	*p*_het_^d^
Age at diagnosis							
<50 years^e^							
ER+/PR+							
Cases/controls	82/70	25/35	13/15				
RR (95% CI)	Ref.	0.48 (0.23-0.98)	0.53 (0.17-1.69)	0.14	0.48 (0.18-1.28)		
ER+							
Cases/controls	99/87	27/40	17/16				
RR (95% CI)	Ref.	0.49 (0.25-0.96)	0.79 (0.30-2.09)	0.32	0.64 (0.26-1.54)		
≥50 years							
ER+/PR+							
Cases/controls	260/242	272/273	277/294				
RR (95% CI)	Ref.	0.90 (0.69-1.18)	0.86 (0.64-1.16)	0.47	0.89 (0.65-1.22)	0.02	0.53
ER+							
Cases/controls	460/423	492/504	527/552				
RR (95% CI)	Ref.	0.87 (0.71-1.06)	0.85 (0.68-1.06)	0.24	0.87 (0.68-1.10)	0.02	0.72
ER–/PR–							
Cases/controls	58/71	71/90	95/63				
RR (95% CI)	Ref.	1.15 (0.65-2.02)	2.54 (1.35-4.77)	0.02	2.21 (1.11-4.40)		0.30
ER–							
Cases/controls	101/119	103/112	123/96				
RR (95% CI)	Ref.	1.21 (0.77-1.92)	1.90 (1.17-3.09)	0.04	1.76 (1.03-3.01)		0.62

Conditional logistic regression models adjusted for ages at menarche (≤12, 13, 14, ≥15, missing), menopause (<44, 44-47, 48-50, 51-52, 53-54, ≥55, missing), first full-term pregnancy (no FTP, <25, 25-30, ≥30, missing), number of full-term pregnancies (0, 1, 2, ≥3, missing), and BMI (kg/m^2^, continuous)

^a^ *p*_trend_ based on log2-transformed continuous variable

^b^RR for a one-unit change in log2-transformed OPG

^c^ *p*_heterogeneity_ comparing ER+/PR+ to ER–/PR– and ER+ to ER– subtypes, based on RRlog2

^d^ *p*_heterogeneity_ comparing age at diagnosis <50 to 50+ years based on RRlog2

^e^Age at diagnosis results limited to ER+/PR+ and ER+ cases given *n* = 34 ER–/PR– cases; *n* = 59 ER– cases in this subgroup

We observed no evidence for effect modification by other reproductive and lifestyle factors (e.g., ever full-term pregnancy, OC use, age at menopause (postmenopausal women) or by levels of endogenous hormones (e.g., estradiol, progesterone, prolactin; data not shown), with the exception of suggestive heterogeneity by prolactin concentrations for ER– disease (prolactin < median, RR = 0.78 [0.23–2.69]; ≥ median, RR = 2.98 [1.23–7.23]; *p*_het_ = 0.05) and heterogeneity by testosterone concentrations for ER+ disease (testosterone < median, RR = 0.35 [0.15–0.79]; ≥ median, RR = 1.05 [0.52–2.13]; *p*_het_ = 0.04). Finally, results were similar in sensitivity analyses restricted to women diagnosed >2 years after blood collection. OPG demonstrated high within-person reproducibility, in terms of the relative ranking of study participants, over 1 year (*r* = 0.85) and 14 years (*r* = 0.75).

## Discussion

Higher serum osteoprotegerin (OPG) concentrations were associated with an increased risk of ER– breast cancer, and a modest, suggestive reduction in risk of ER+ tumors, in this large investigation. With a total of 2008 breast cancer cases with documented ER status, this is the first large-scale prospective study to address circulating OPG and subsequent breast cancer risk by hormone receptor subtype.

Vik et al. reported an inverse association between OPG concentrations and breast cancer risk in a case-cohort study of more than 6000 women and men (median 13.5-year follow-up) [12]. This Norwegian investigation included only 76 breast cancer cases and thus could not address risk by hormone receptor status. Most likely, however, the majority of breast cancer cases were ER+, as this is the predominant subtype among European women. The suggestive inverse association between OPG and ER+ breast cancer risk observed in the current study lies in the same direction as in this previous investigation, although the association observed in our study is weaker. In two analyses restricted to *BRCA1/2* carriers, higher serum OPG concentrations were associated with lower risk mutation site [22] and lower breast cancer risk (*n* = 18 cases) [23]. *BRCA1/2* mutation status was not available in our study population, and family history of breast cancer is missing for the majority of participants (64%). Therefore, we were unable to evaluate OPG and breast cancer risk restricted to a higher risk population in our study. These three investigations used ELISA assays, while we used an electrochemiluminescence assay. Vik [12] and Oden [23] observed starkly different mean/median OPG concentrations in their two study populations (Vik, reported mean concentration: 3.40 ng/mL; Oden, reported median concentration, 95 ng/mL). Widschwendter et al. [22] did not directly report mean or median OPG concentration. To date, there is no cross-assay standardization protocol for OPG. Thus, between-study comparisons must rely on the within-study relative ranking of participants.

Prior investigations show high levels of OPG protein or mRNA in a variety of breast cancer cell lines [3, 6, 24–27], with lower levels in primary human mammary epithelial cells [6]. Studies of primary breast tumors also showed variable degrees of OPG protein [3, 6, 24, 28–30] or mRNA expression [30–34]. In larger patient series, immunohistochemical analyses [24, 29, 35] indicated OPG protein in 40-45% of invasive primary tumors, an inverse association between OPG expression and tumor grade and stage, and a positive association with ER status [24, 29, 30]. Studies based on mRNA analysis, however, showed a positive [36], a negative [33, 34], or no association [32] between the gene expressions for OPG and ER status. A limited number of studies have examined non-neoplastic mammary tissue. On balance, these investigations observed some level of OPG expression, however, at lower levels than that observed in breast tumor tissue [6, 29, 31, 37]. In a large, multicenter analysis of more than 4500 patients, higher OPG mRNA expression was associated with reduced breast cancer-specific mortality among patients with ER+ tumors (*n* = 1941), but not among patients with ER– disease (*n* = 649) [33]. These observations support a role for OPG in breast tumor development and prognosis, with potential differences between ER- and ER+ disease.

We observed increased risk of ER– (and ER–/PR–) breast cancer among women with higher serum OPG concentrations. OPG binding to TRAIL represents one key mechanism through which OPG could promote breast tumor development, particularly of ER– tumors [8, 38]. TRAIL is highly homologous to tumor necrosis factors (TNFs), and induces apoptosis through specific TRAIL receptors in a diverse range of cell lines, and preferentially in cancer cells [39]. Analyses in breast cancer cell lines have shown that triple-negative cells (i.e., ER–/PR–/HER2–) are especially sensitive to TRAIL-induced apoptosis, whereas sensitivity to TRAIL varies across different HER2+ cell lines, and is systematically absent among ER+ cells [7]. Our study population included only 58 triple-negative cases, and we observed no association in this subgroup. We observed a stronger association between OPG and ER– breast cancer risk among women with relatively high circulating prolactin concentrations. Prolactin inhibits TRAIL-induced apoptosis in hormone (androgen)-insensitive PC3 prostate cancer cells [40]. To our knowledge there are no data on prolactin and TRAIL in breast cancer; however, prolactin inhibition has been shown to increase apoptosis in breast cancer cell lines [41], and prolactin interferes with the apoptotic effect of cisplatin chemotherapy [42].

We observed a suggestive inverse association between OPG and ER+ breast cancer. OPG may reduce breast tumor development through binding to RANKL. In addition to regulating bone turnover, RANK and RANKL mediate progesterone- and prolactin-induced changes in the mammary gland, including the expansion of mammary epithelial stem cells [43], proliferation of the mammary epithelium [44, 45], and the development of a lactating mammary gland during pregnancy [46]. Furthermore, experimental animal studies have demonstrated that increased expression of RANKL in PR+ luminal epithelial cells, following autocrine and paracrine stimulation of RANK signaling in both receptor positive and negative epithelial cells, is a mechanism mediating progestin-driven mammary carcinogenesis [43, 44, 47, 48]. Circulating OPG may modulate these effects, with one investigation showing increased mammary epithelial proliferation after intravenous administration of soluble RANKL (sRANKL), and inhibition of proliferation by the administration of OPG, in an animal model [44]; in vitro investigations of administered RANKL and OPG in human breast tumor samples show similar results [49]. It is conceivable that OPG inhibits the development of ER+/PR+ breast tumors by interfering with the growth-promoting effects of RANKL, which depend on PR-mediated local synthesis.

Breast cancer risk increases with higher lifetime number of menstrual cycles, an indicator for cumulative exposure to luteal phase progesterone levels, and is also increased among postmenopausal women using combined estrogen-plus-progestin HT [50–52]. Interestingly, RANKL expression in both normal and malignant breast tissue is also increased during the luteal phase of the menstrual cycle [53] and in combined HT users [54]. We observed no indication that the association of serum OPG concentrations with breast cancer risk varied between tumors diagnosed before or after age 50 (the median age at menopause in EPIC), by age at menopause, or between users and non-users of HT.

While several tissue types produce OPG, bone marrow cells and vascular endothelial cells may be the predominant sources of circulating OPG [55–57]. Estrogens, vitamin D3, and growth hormone promote OPG synthesis in vitro (e.g., by osteoblasts) [55, 58, 59]. Limited cross-sectional data in women support no, or only modest, associations between circulating hormones (e.g., estradiol [60], progesterone [22]) and OPG in healthy women. As in other recent studies [22, 61], we observed no systematic variation of serum OPG concentration with phase of menstrual cycle or with menopausal status (after adjustment for age at blood collection) [62], and a gradual rise of OPG levels with increasing age [58, 62, 63]. Prior studies provide mixed results on whether postmenopausal HT use is associated with OPG [64–66]. We observed somewhat higher OPG concentrations among postmenopausal women using HT, with no differences by HT type (i.e., opposed vs. unopposed estrogens). In subgroup analyses, where additional measurements of endogenous hormones were available, we observed no correlations between OPG and DHEAS, androstenedione, testosterone, estradiol, IGF-I, or prolactin.

## Conclusions

In summary, recent research has revealed intriguing parallels in the roles for OPG and the RANK/RANKL axis in bone metabolism and in mammary gland physiology. Our large-scale investigation is the first to document potentially diverse associations of OPG with risks of ER– or ER+ breast cancer. There is increasing interest in denosumab — a humanized antibody that blocks RANKL signaling, which is used routinely to treat osteoporosis — as a drug for preventing breast cancer recurrence among women with early stage breast cancer and primary prevention in high risk women [67]. Although the role of OPG in breast cancer is complex, due to its possible interactions with both TRAIL and RANKL, our data may provide indirect insight into the possible benefits or risks potentially associated with treatments targeting the RANK/RANKL system.

## Additional file

Additional file 1: Table S1. Characteristics of the within-person reproducibility sample (*n* = 221) at baseline (1994–1998) and follow-up (2000–2003). **Table S2.** Correlations between OPG and select hormones among controls: EPIC cohort nested case-control study. **Figure S1.** Evidence of potential non-linearity of the association between OPG and ER+ breast cancer among women premenopausal at blood collection from spline regression model: EPIC cohort nested case-control study. (DOCX 84 kb)

## Abbreviations

BMI: Body mass index
CI: Confidence interval
CV: Coefficient of variation
EPIC: European Prospective Investigation into Cancer and Nutrition
ER: Estrogen receptor
FTP: Full-term pregnancy
HT: Hormone therapy
IARC: International Agency for Research on Cancer
LRT: Likelihood ratio test
OC: Oral contraceptive
OPG: Osteoprotegerin
PR: Progesterone receptor
QC: Quality control
RANK: Receptor activator of nuclear factor kappa-B
RANKL: Receptor activator of nuclear factor kappa-B ligand
RR: Relative risk
sRANKL: Soluble RANKL
TNF: Tumor necrosis factor
TRAIL: TNF-related apoptosis-inducing ligand
