# Supplementary material for: Osteoprotegerin and breast cancer risk by hormone receptor subtype: a nested case-control study in the EPIC cohort
Source: BMC Med. 2017 Feb 8;15:26. doi: 10.1186/s12916-017-0786-8 (PMC5297136; doi:10.1186/s12916-017-0786-8)

| **Table S1. Characteristics of the within-person reproducibility sample (n=221) at baseline (1994-1998) and follow-up (2000-2003)** | | |
| --- | --- | --- |
|  | ***Baseline*** | ***Year 14*** |
| ***N (%) or Median (10-90^th^ percentile)*** | |  |
| Age, years | 51 (37-60) | 65 (52-74) |
| Age at menarche, years | 13 (11-15) | -- |
| Premenopausal | 93 (42.1%) | 11 (5.0%) |
| Perimenopausal | 38 (17.2%) | 1 (0.4%) |
| Postmenopausal | 90 (40.7%) | 209 (94.6%) |
| HT use at blood collection* | 61 (67.8%) | 25 (11.3%) |
| Age at menopause, years* | 48 (45-53) | 50 (45-54) |
| Completed Term Pregnancy | 184 (83.3%) | -- |
| Age at first term pregnancy^†^, years | 26 (21-33) | -- |
| BMI, kg/m^2^ | 24 (21-30) | 25 (21-31) |
| **Among postmenopausal women*  *^†^ Among women with completed term pregnancy* | | |

| **Table S2. Correlations between OPG and select hormones among controls: EPIC cohort nested case-control study** | | | | | | | | | | |
| --- | --- | --- | --- | --- | --- | --- | --- | --- | --- | --- |
|  | **Estradiol** | **Estrone** | **Testosterone** | **DHEAS^*^** | **SHBG^*^** | **Progesterone** | **Prolactin** | **IGF1^*^** | **BMI^*^** | **Age*^†^*** |
|  |  |  |  |  |  |  |  |  |  |  |
| **Premenopausal, n** | 333 | 125 | 420 | 158 | 420 | 297 | 462 | 333 | 462 | 462 |
| **OPG** | -0.01 | -0.14 | -0.07 | -0.03 | 0.10 | -0.03 | -0.10 | -0.1 | -0.01 | 0.11 |
| *p* | *0.80* | *0.15* | *0.18* | *0.74* | *0.04* | *0.67* | *0.03* | *0.05* | *0.95* | *0.02* |
|  | | |  |  |  |  |  |  |  |  |
| **Postmenopausal, no HT, n** | 402 | 232 | 401 | 233 | 401 |  | 791 | 402 | 791 | 791 |
| **OPG** | 0.03 | -0.03 | 0.01 | -0.05 | -0.01 | ^ǂ^ | -0.14 | -0.06 | 0.02 | 0.34 |
| *p* | *0.52* | *0.61* | *0.83* | *0.46* | *0.91* |  | *<0.01* | *0.27* | *0.64* | *<0.01* |
|  |  |  |  |  |  |  |  |  |  |  |
| **Postmenopausal, using HT, n** |  |  |  |  |  |  | 755 |  | 755 | 755 |
| **OPG** | *^ǂ^* | *^ǂ^* | *^ǂ^* | *^ǂ^* | *^ǂ^* | *^ǂ^* | -0.05 | *^ǂ^* | -0.03 | 0.23 |
| p |  |  |  |  |  |  | *0.21* |  | *0.41* | *<0.01* |
| *Adjusted for: age at blood collection, fasting status, time of day of blood collection, study center (country)* | | | | | | | | | | |
| **DHEAS:* *Dehydroepiandrosterone-Sulfate; SHBG: Sex Hormone Binding Globulin; IGF1: Insulin-like Growth Factor I; BMI: Body Mass Index (kg/m^2^)* | | | | | | | | | | |
| ***^†^****In years, at blood collection; not age-adjusted* | | | | | | | | | | |
| *^ǂ^Not measured* | | | | | | | | | | |

**Figure S1.** Evidence of potential non-linearity of the association between OPG and ER+ breast cancer among women premenopausal at blood collection from spline regression model: EPIC cohort nested case-control study
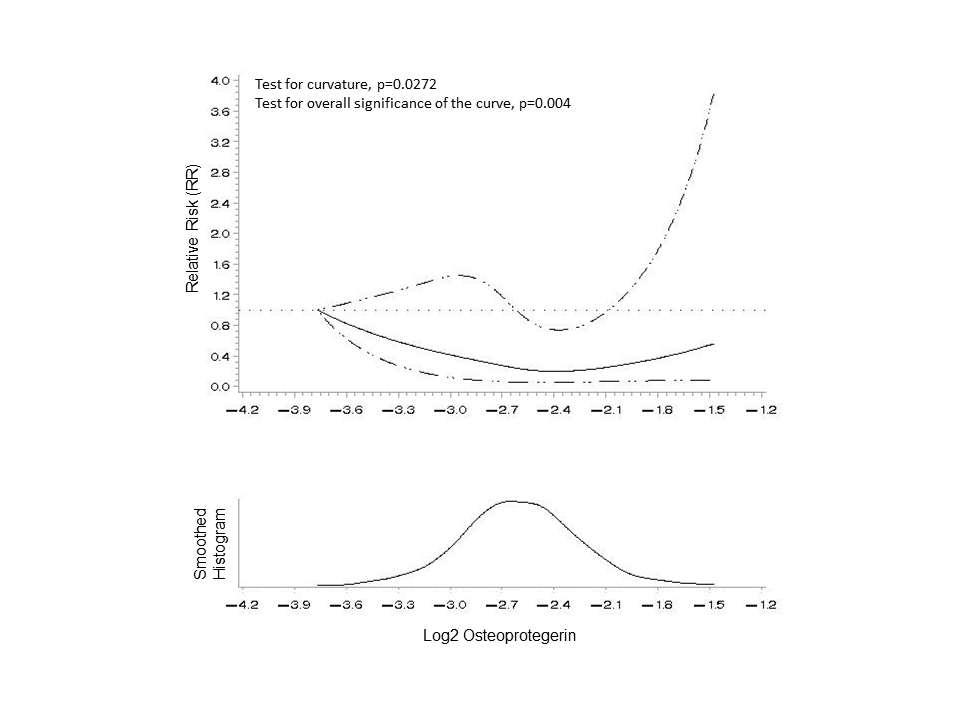

Supplement: Additional file 1: Table S1. — Characteristics of the within-person reproducibility sample (n = 221) at baseline (1994–1998) and follow-up (2000–2003). Table S2. Correlations between OPG and select hormones among controls: EPIC cohort nested case-control study. Figure S1. Evidence of potential non-linearity of the association between OPG and ER+ breast cancer among women premenopausal at blood collection from spline regression model: EPIC cohort nested case-control study. (DOCX 84 kb) [file 12916_2017_786_MOESM1_ESM.docx]
